# Measuring the tilt and slant of Chinese handwriting in primary school students: A computerized approach

**DOI:** 10.1371/journal.pone.0223485

**Published:** 2019-11-04

**Authors:** Monica M. Q. Li, Howard Leung, Tim M. H. Li, Cecilia W. P. Li-Tsang

**Affiliations:** 1 Department of Computer Science, City University of Hong Kong, Kowloon Tong, Hong Kong; 2 Department of Rehabilitation Sciences, The Hong Kong Polytechnic University, Hung Hom, Hong Kong; University of Toronto, CANADA

## Abstract

**Background:**

“Horizontal strokes should be level and vertical strokes should be straight” is a common guideline in the teaching of Chinese handwriting. Measuring deviations in level horizontal and straight vertical strokes in students’ Chinese handwriting is usually assessed manually. However, this task is time-consuming and may have inconsistent outcomes when judged by different people. In this paper, we aim to formulate a method to automatically evaluate the tilt and slant degrees of students’ Chinese handwriting using digital handwriting tablets. Furthermore, we analyze the relationship between the tilt and slant features of students’ Chinese handwriting and other demographic and handwriting features.

**Methods:**

Five hundred and ninety-one primary school students from grades 1 to 6 were recruited in Hong Kong. Before the assessment, a grid paper was attached to a digital handwriting tablet. The participants were then asked to copy 90 Chinese characters from a template to the grid paper. Their handwriting processes were recorded as two-dimensional points and then analyzed. The tilt and slant of the students’ handwriting were calculated based on the inclination level of their horizontal and vertical strokes. Linear regressions between slant/tilt degree of the manuscripts and other handwriting features were performed. The students’ demographic information was also explored.

**Results:**

Slant was found to be significantly correlated to Gender (p < 0.001) and tilt×standard deviation of pen pressure (p < 0.001). Tilt was found to be significantly correlated to ground time (p < 0.001), slant (p < 0.001) and slant×special education need (p = 0.021).

**Conclusions:**

Our results demonstrate the relationship between slant, tilt and Chinese handwriting performance in primary school children. Slant and tilt can be adopted as an indicator in students’ special education need diagnosis, as tilt level in the students’ Chinese handwriting was related to ground time and slant× special education need, while slant is related to tilt×standard deviation of pen pressure and female students. These findings may also inspire ways to increase special education need students’ writing speed.

## Introduction

The inclination of strokes in characters is a prominent feature in individual handwritings, as the inclination of strokes determines the whole structure of characters. However, the definition and quantification of inclination are still blurred and incomplete for different kinds of strokes, especially in Chinese handwriting. This study aims to quantify the inclination of strokes in Chinese handwriting and evaluate whether the inclination of strokes is related to other handwriting and demographic features of students.

If we refer to a similar concept in English handwriting, the slant of cursive English writing is defined as the angle of inclination of the axis of letters relative on the baseline [1], is an important writing feature. Difficulties with Western letter slant affect handwriting legibility, thereby constituting poor handwriting [2]. Poor writers have difficulty achieving proper slant in their handwriting [3]. Some Western writing assessment systems include slant in their assessment criteria. The Test of Legible Handwriting [4] separates left and right slanted writing to measure the legibility of handwriting for children in grades 2 to 12. The Children’s Handwriting Evaluation [5] considers slant in its quality score rating to measure the fluency and quality of children’s handwriting from grades 3 to 8.

In the Chinese handwriting system, the concept of slant also exists in manuscripts. “Horizontal strokes should be level and vertical strokes should be straight” is a common guideline in the teaching of Chinese handwriting [6]. However, measuring inclination in Chinese should be done in two directions instead of just one, as in the Western writing system. Specifically, cursive Latin letters do not contain the long horizontal strokes that appear frequently in Chinese characters. Furthermore, Chinese characters are more complicated and have more strokes in different directions, as shown in Table 1 [7]. As the concept of slant in Latin letters refers to the dominant angle of near-straight downward strokes with respect to the horizontal [8], a similar measurement of inclination in horizontal strokes is essential in assessing Chinese handwriting.

**Table 1:** Basic Chinese character strokes.

Stroke	English title	Pinyin and Chinese title	Meaning in Chinese	Description
ヽ	Dot	*Diǎn*, 點	Dot	Tiny dash, speck
ㄧ	Horizontal	*Héng*, 橫	Horizontal	Rightward stroke
〡	Vertical	*Shù*, 豎	Vertical	Downward stroke
㇀	Upward horizontal	*Tiāo*, 挑	Rise	Flick up and rightward
㇏	Press	*Nà*, 捺	Press down	Falling rightward (fattening at the bottom)
ノ	Throw	*Piě*, 撇	Throw away	Falling leftward (with a slight curve)

**Fig 1:**
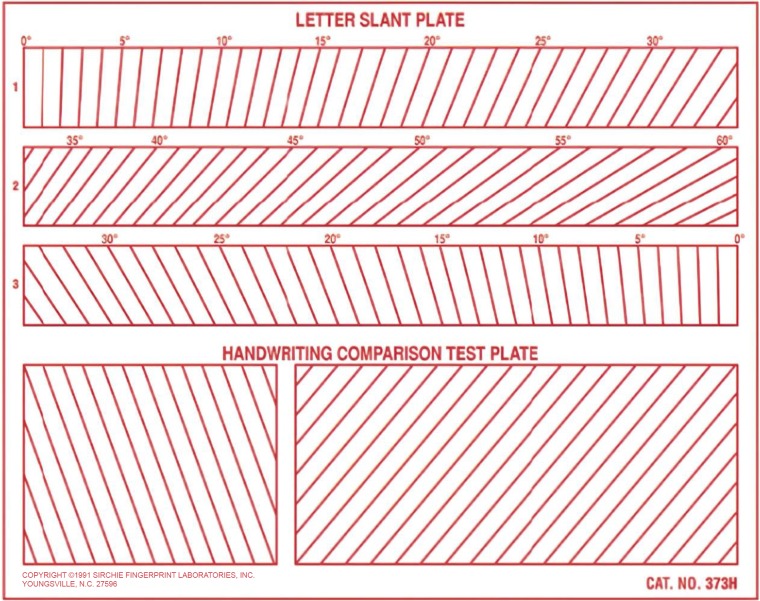
Slant plate.

A comprehensive Chinese handwriting examination study at the Forensic Science Division of the Hong Kong Government Laboratory identified inclination features as measurable parameters [9]. In this series of studies, slant was referred to as the angle of inclination of vertical stroke relative to the axis of the character and tilt was referred to as the angle of inclination of the horizontal stroke relative to the line of writing. The researchers measured the slant of a vertical stroke using a slant plate, an instrument (SKU: SI373H) produced by the American Society of Questioned Document Examiners, as shown in Fig 1 [10]. Slant plates can also be used to measure the tilt of a horizontal stroke. A protractor was also used. As some strokes were not straight, subjective judgments were made regarding the best fit for the measurements. Accordingly, using this measurement method to evaluate many children’s manuscripts would have been both tedious and subjective.

Regarding computerized methods of measuring the slant degree of Chinese characters, several typical algorithms have been created. For instance, several studies have adopted the Hough transform in the skew correction process [11][12][13][14][15][16]. This method maps shapes on a Cartesian coordinate system into a polar coordinate. After the transform, every straight line in the original coordinate system is represented by a point, and straight lines with the same slope share the same abscissa. Therefore, the overall slant and tilt of an image can be determined by finding the abscissa with the most points. However, this method mainly applies to printed characters, as the directions of certain types of stroke are identical. Handwritten characters have various slant levels from the same types of stroke. Thus, the application of this method to manuscripts is limited.

Direction-based pattern extraction has also been conducted in some studies [17][18][19], establishing a four- or eight-directional-based pattern to define standard stroke types. Such stroke types include horizontal, vertical, press, and throw strokes, and the same stroke shapes produced in the opposite direction of writing. Whole characters or strokes are then divided into standard types of strokes for further analysis. This practice is suitable for assessing the manuscripts of adult writers, who already understand and implement the concept of strokes. However, it is difficult to apply in assessments of children’s handwriting, as some young students lack the concept of strokes and produce strokes that are scrawled arbitrarily and even concatenated cursively. This consequently affects the accuracy of the extraction. Another drawback of this practice is that the original slant and tilt degree of a stroke is lost in the extraction. Therefore, the results can only show the number of strokes under each type without analyzing the movement inside of each stroke.

Classic slant judgment methods also include Bozinovic and Srihari’s method [20], Kimura, Shridhar, and Chen’s method [21], and their modifications [22]. These methods include all non-horizontal strokes in evaluating writing slant and are fit for Western writing styles. Nevertheless, some strokes with inherent slopes (e.g., “press” and “throw” in Table 1) should not be considered in the slant assessments of Chinese handwriting. As such, these methods are not applicable to this study.

We propose a method of measuring the slant and tilt of primary school students’ handwriting. It is accomplished by estimating the mean slant and tilt based on the average slope of each movement vector of horizontal and vertical strokes during the handwriting process. The students’ handwriting processes are accurately recorded in graphic tablets, similar to other research [23][24]. However, our proposed method views the students' handwriting in a microscope way directly while other methods requiring transform or projection often result in information loss. Our calculation results are presented accordingly.

The modelling of the slant/tilt to other handwriting and demographic features of students corresponds to the modelling of the two following hypotheses:

H_0_—Slant and tilt are not related to any handwriting and demographic feature of students.

H_1_—Slant or tilt are related to at least one handwriting and demographic feature of students.

We can also model the two hypotheses under a mathematical form. This can be accomplished using classical regression modelling. H_0_ will serve as the null hypothesis or the default hypothesis:

$$Slant=\sum_{i=1}^{n}\beta_{si}x_{i}+\beta_{s0}+\varepsilon_{s}\;\text{and}\;Tilt=\sum_{i=1}^{n}\beta_{ti}x_{i}+\beta_{t0}+\varepsilon_{t}$$

Individual items of *x*_*i*_ are listed in Table 2.

H_0_: *β*_*si*_ = *β*_*ti*_ = 0 *for all i* = 1 *to n*
H_1_: *β*_*si*_ ≠ 0 *or β*_*ti*_ ≠ 0 *for at least one i* = 1 *to n*

**Table 2:** Handwriting performance parameters to be entered into the linear regression model.

Factor name	Explanation
Age	The age of a student at the time of the test
Gender	Nominal value of a student’s gender. 0 represents male and 1 represents female.
Grade	The grade of a student at the time of the test
Handedness	Nominal value of a student’s handedness. 0 represents right and 1 represents left.
SEN	Nominal value of special education needs. 0 represents typical developed students and 1 represents students with special education needs.
Ground_Time	The total time when the pen touched the paper during the assessment
Air_Time	The total time when the pen was in the air during the assessment
Air_Ground_Ratio	Air_Time divided by Ground_Time
Speed	The average characters written by the student in a minute
SD_Time_Per_Word	The standard deviation of the time used on every character during the assessment
Pressure	The average pressure between the pen and paper during the assessment
SD_Pressure	The standard deviation of the pressure for each data point during the assessment
Out_Grid	The ratio of characters written out of grid among the 90 characters

The correlations between slant/tilt and other physical handwriting features were then analyzed, as well as students’ demographic background, so that we can identify potential applications of slant and tilt features of students’ handwriting.

## Methods

### Participants

The research team conducted participant recruitment from 2017 to 2018. Data were collected from six local primary schools in four regions—New Territories (2 schools), Kowloon (2 schools), Hong Kong Island (1 school), and the Outlying Islands (1 school)—according to the proportionality of the total population in Hong Kong. A class from each primary school was randomly chosen. The students from the chosen class fulfilling the following inclusion criteria were invited to participate in the study: (a) able to speak Cantonese and (b) able to write in traditional Chinese. The students were categorized into two groups: (1) typically developing students and (2) students with special education needs (SEN) who had confirmed diagnosis of neurodevelopmental disorders made by pediatrician or psychiatrist using International Statistical Classification of Diseases and Related Health Problems 10th Revision (ICD-10) or Diagnostic and Statistical Manual of Mental Disorders, 5th Edition (DSM-5) in Hong Kong and recorded by the Education Bureau. Students attending special schools or physically handicapped were excluded from this study.

All of the parents and students were informed about the voluntary basis of their participation and gave written informed consent. No remuneration was given to any of the study participants. A handwriting report on each participant’s performance was provided by the end of the assessment. Ethical approval was obtained from the Human Subjects Ethics Committee of the Hong Kong Polytechnic University. Ultimately, a total of 591 subjects were recruited and served as the foundation for further experiment and analysis. The response rate was 67%.

### Measures

#### Digit Note

**Fig 2:**
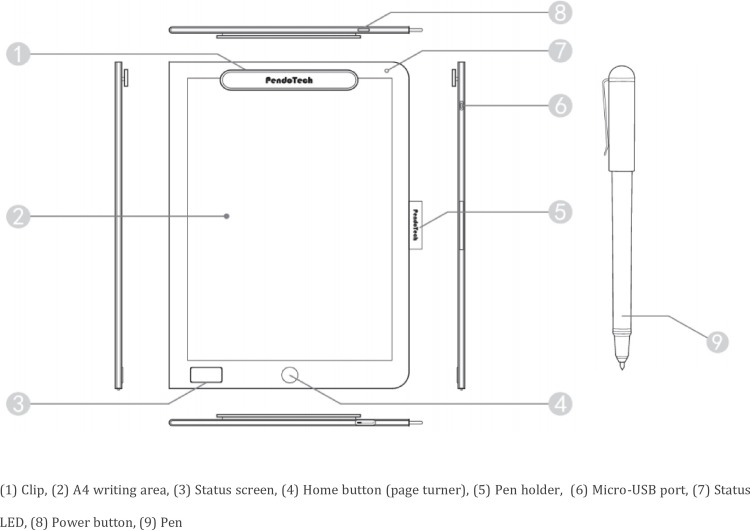
Digital handwriting tablet (Digit Note PH-1410, FCC ID: 2AI7G-PH1410) [25].

The handwriting data used in this study were recorded with Digit Note (Type PH-1410) handwriting tablets developed by PendoTech, as shown in Fig 2. Each of the tablets was 248.5×344×10.0 mm^3^ with a 140×22×17 mm^3^ clip on the top edge. They each contained an A4 writing area 2 mm lower than the outer border so that the A4 paper would not move from the tablet during the writing process. A pen (length: 133.4 mm, diameter: 9.5 mm, weight: 10.8 g) was attached to the tablet and contained a black ink cartridge, similar to a normal rollerball pen.

During the writing process, the position of the pen tip was recorded by the tablet every 20 milliseconds, creating 50 data points per second. Simultaneously, the pressure between the pen tip and the tablet was recorded with each data point. If the pen tip did not make contact with the tablet at the time of recording, the pressure was recorded as zero. All of the data of one sample were stored in an array of points, each of which had three properties: the x-coordinate, the y-coordinate, and the pressure of the point. The x- and y-coordinates had a precision of 0.01 mm.

After data collection, the writing data were uploaded via Bluetooth to the computer. Each stroke was separated based on the pen pressure, with the non-zero pressure indicating the process of writing a stroke and the zero pressure indicating air movements between two strokes. All of the non-zero pressure points were classified as the real data part and depending on the time used to write that specific stroke. Each array represented one stroke.

**Fig 3:**
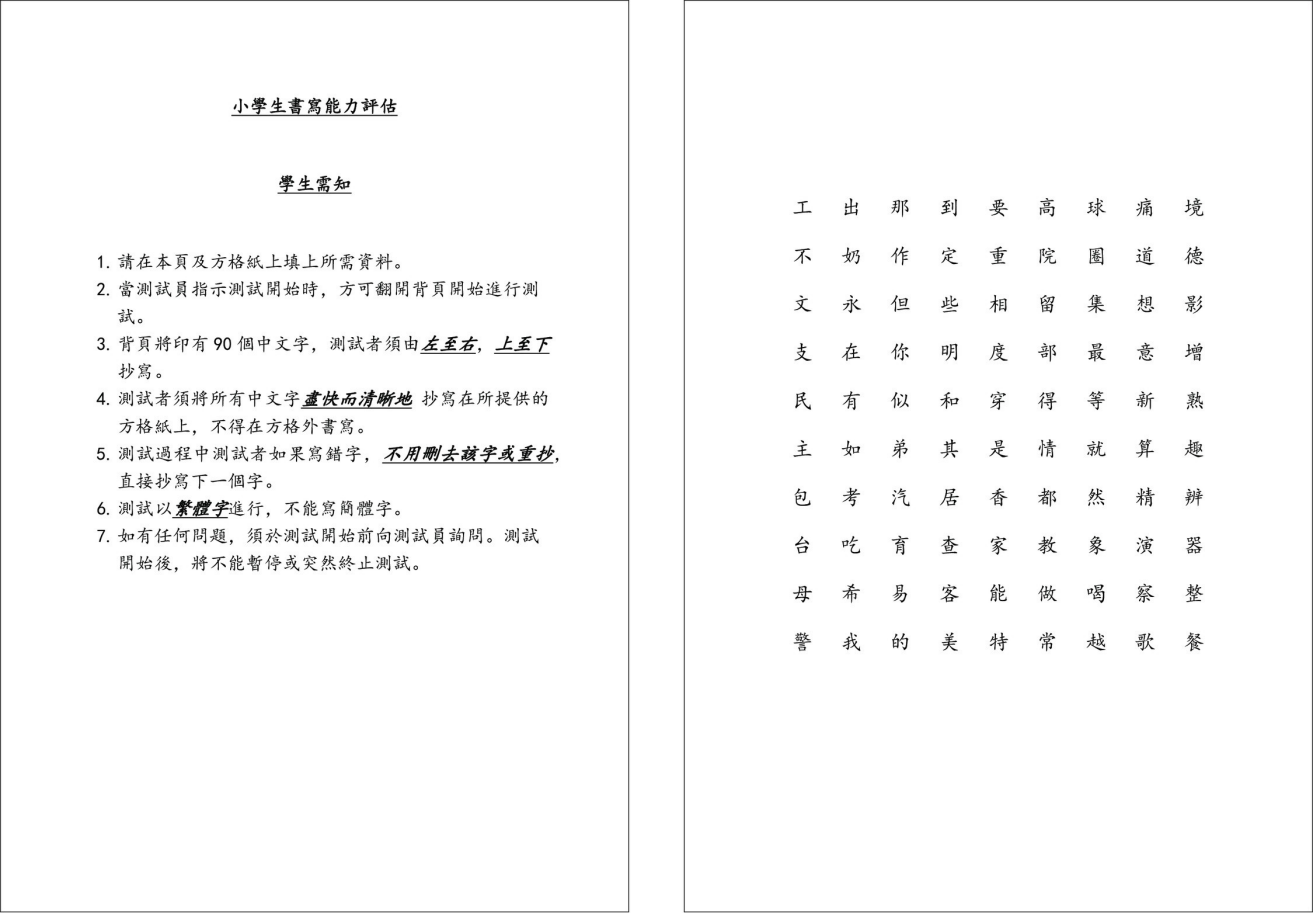
Handwriting assessment template.

We then separated the whole array of all the strokes into individual characters based on the relevant positions and writing sequences of the strokes. If a student completed the whole copying task, 90 characters in Fig 3 were identified from the writing data, such that the real data part contained 90 objects.

Finally, the speed of the students’ handwriting (Speed), the SD of the writing time used per character (SD of writing time per character), the average pressure during the writing process (Pressure), the SD of pressure during the writing process (SD of pressure), and the percentage of characters written out of grid (Out of grid) were calculated.

### Procedure

#### Handwriting assessment

After the participants had been selected, a handwriting assessment was conducted using an electronic tablet. A 9×10 grid was printed on an A4 paper, with single square boxes of 1×1 cm^2^. The A4 papers were prefixed on the tablets before distribution to the students for the assessment.

**Fig 4:**
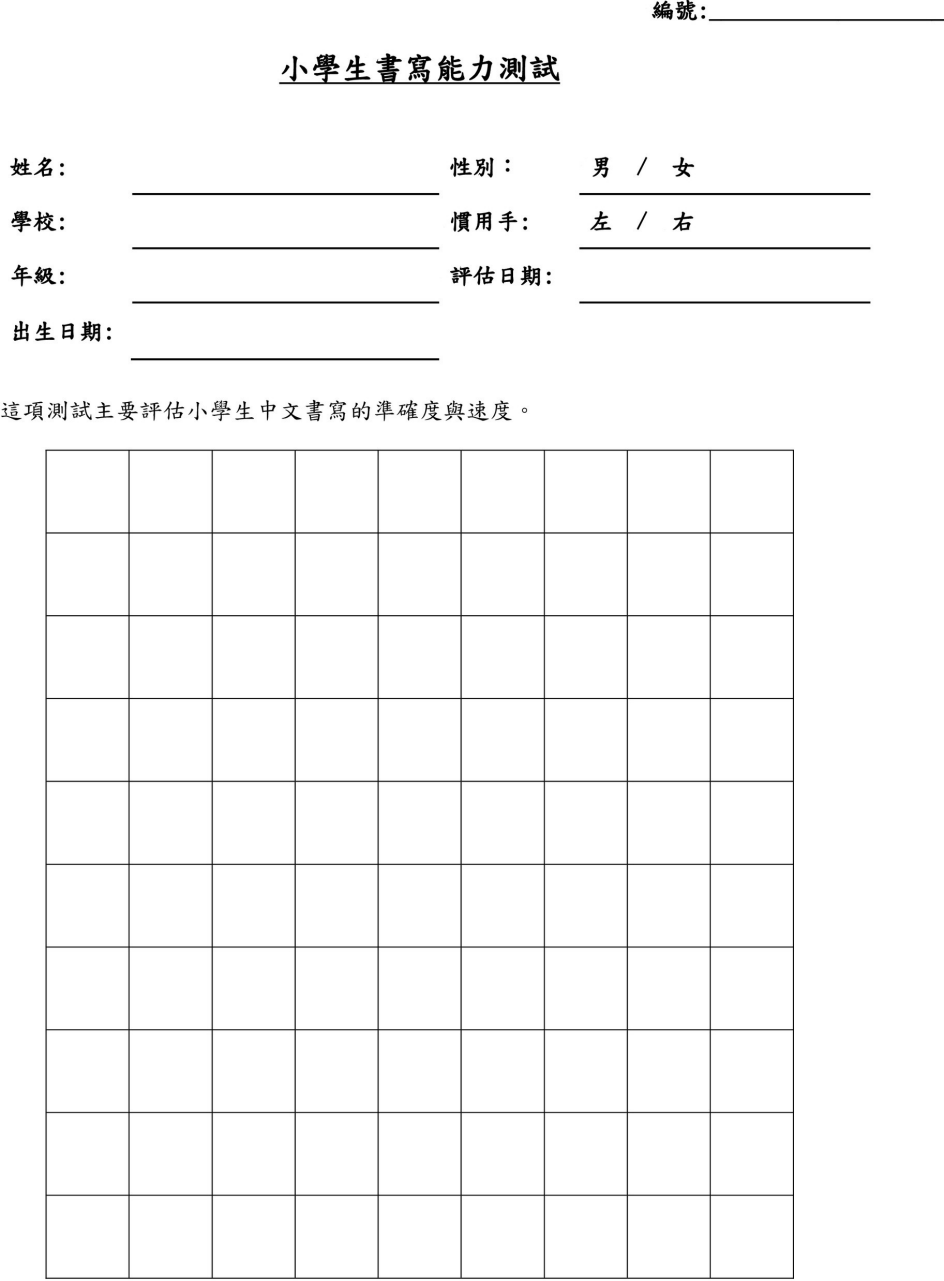
Handwriting assessment grid paper.

During the handwriting assessment, the students were instructed to copy 90 traditional Chinese characters (Fig 3) onto a test paper (Fig 4). These characters, which did not form a meaningful text when combined, were used as a standardized sample for students to copy during the assessment. A copying task instead of a generative writing process was implemented because this experiment aimed to assess the students' handwriting features apart from their thinking process in generative writing. These characters were chosen from the “Chinese, Taiwanese, and Hong Kong Modern Chinese Corpus” and based on the criteria of various distribution and difficulty levels and the frequency of writing in grades 1 to 6 [26]. The 90 Chinese characters were selected to represent characters with different stroke radicals and character structures, including different “up-and-down,” “left-and-right,” and “in-and-out” forms. Only characters with the same format in traditional and simplified Chinese were included to prevent students who had learned simplified Chinese from mixing it up with traditional Chinese.

#### Tilt and slant assessments via Python libraries

After the handwriting data were extracted, all of the coordinates of individual students’ handwriting sample points were stored in separate files identified by the students’ assessment IDs. All of the coordinates of each sample were imported to Pandas DataFrame, along with their stroke and character numbers. To visualize the results, all of the points in the data frame were printed onto a PNG image using Python Imaging Library (PIL) to check whether the coordinates matched the students’ handwriting results.

**Fig 5:**
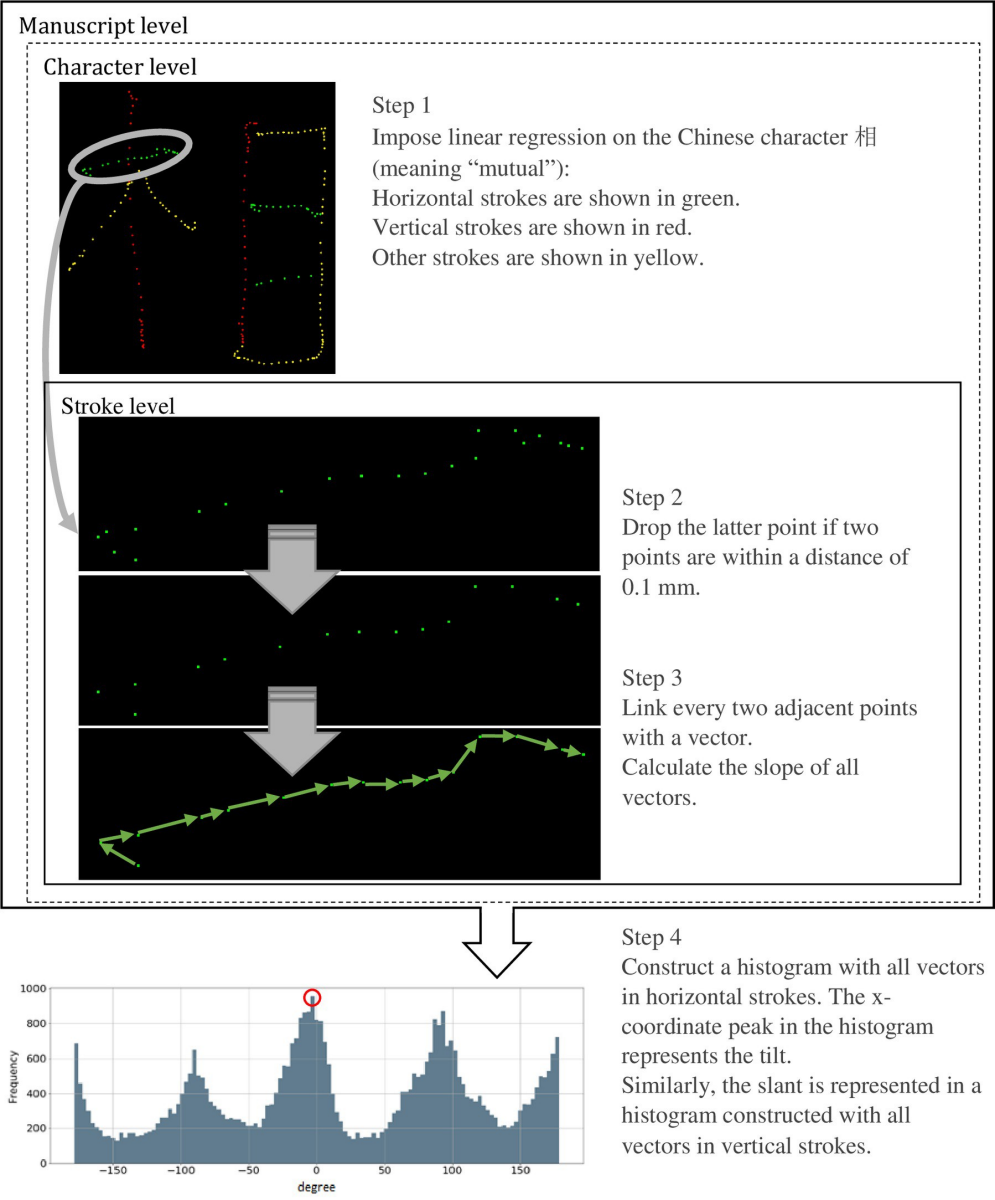
Tilt and slant assessment.

Linear regression was performed on each stroke via the Scikit-learn Package based on points from each stroke to examine the stroke’s overall slant or tilt angle. If the angle of the regression line was between –30° and 30°, the stroke was classified as a horizontal stroke (green points in Fig 5). If the angle of the regression line was between 60° and 120° or -120° and -60°, the stroke was classified as a vertical stroke (red points in Fig 5). If the angle of the regression line fell outside of the intervals above, the stroke was classified as neither a horizontal nor a vertical stroke (yellow points in Fig 5). The horizontal and vertical parts of a yellow point stroke were not processed further, as they did not influence the tilt and slant results on the whole manuscript level.

**Fig 6:**
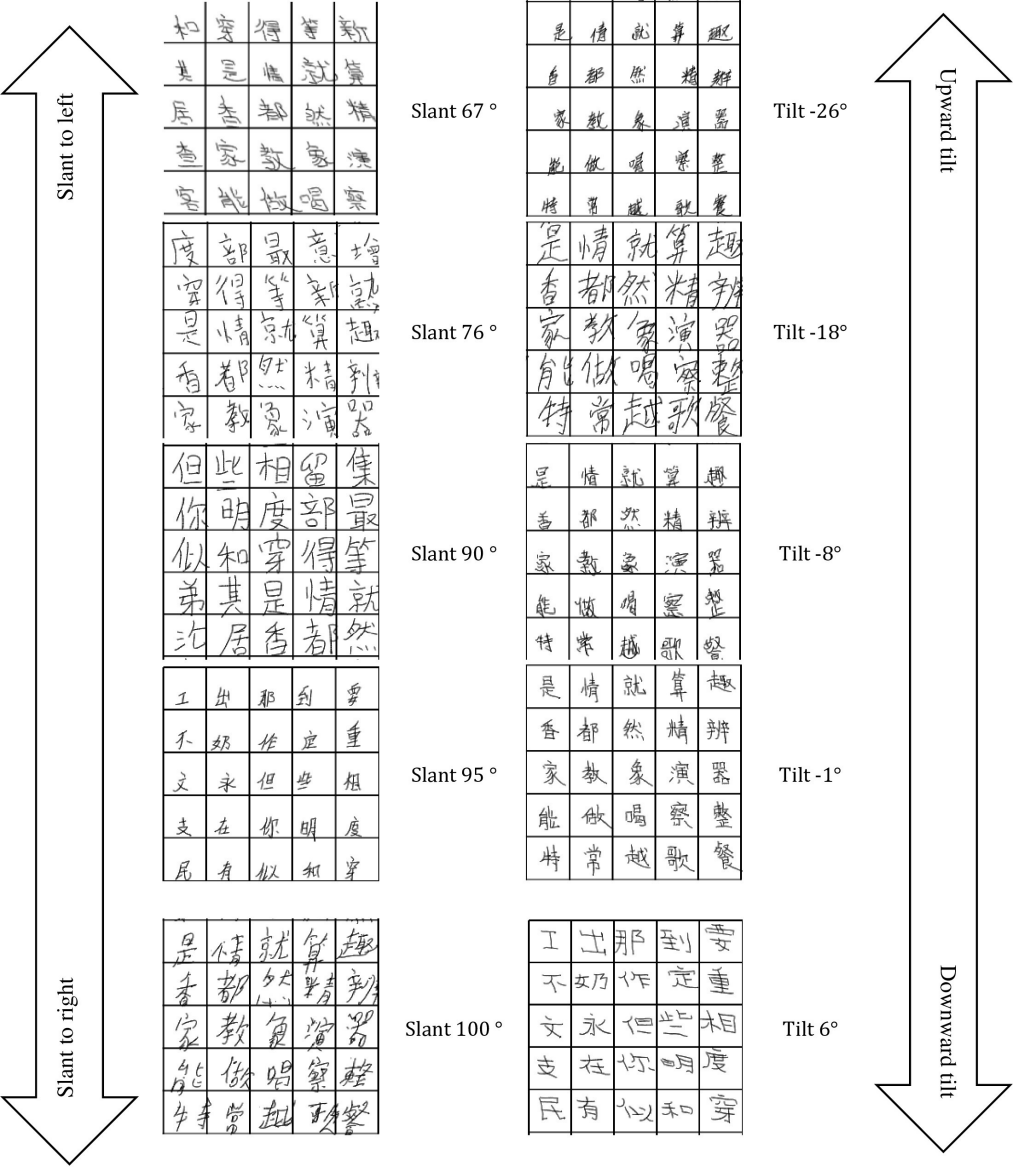
Tilt and slant assessment results.

With a single stroke, if two adjacent points on one stroke were within a distance of 0.1 mm, the latter point was dropped and the next point was examined via the same rule to ensure that the distances between all adjacent points in the data frame were over 0.1 mm. This re-sampling operation removed the noise caused by abrupt directional changes between two nearby adjacent points, especially at the start or end of a stroke. A vector was then formed between every two adjacent re-sampled points on a stroke. The angle of the vector was calculated and stored in the data frame. The angle distribution of all of the vectors from vertical (horizontal) strokes was plotted in a histogram, with the peak representing the overall slant (tilt) degree of the writer. This tilt and slant judgment was used to assess the handwriting of the 591 participating students. Some of the assessment outcomes are shown in Fig 6.

### Statistical analysis

The means and SDs of all of the SEN and typically developing students’ handwriting were calculated (Table 3). The mean tilt and slant of the typically developing students’ were consistent with adult writers’ writing data and the standard computer font, Kai (used in the writing template and teaching practices). A tilt of -9° was also consistent with typical adult manuscripts written under the same experimental conditions and the average value shown in the study of Leung, Cheung, Fung, and Cheung [27].

**Table 3:** Means and standard deviations of the students’ handwriting.

	Typically developing students		SEN students	
	Tilt	Slant	Tilt	Slant
Mean	-9.1	90.3	-7.3	90.7
SD	6.7	4.5	6.6	4.5

Of the 591 students included in the analysis, 507 were typically developing students and 84 were SEN students. The age distribution of different grades and detailed demographic characteristics are shown in Tables 4, 5 and 6. There were no significant differences in demographic characteristics across the different groups of grades, as shown by the chi-square test results. The p-values for the chi-square tests between the typically developing group and the SEN group were < 0.001 for gender and 0.887 for handedness. There were significantly more boys than girls in the SEN group.

**Table 4:** Demographic characteristics of typically developing students.

		Typically developing students (n = 507) n(%)						p-value
Grade		1	2	3	4	5	6	
N		114	94	76	76	76	71	
Gender	Male	58(11.4)	47(9.3)	40(7.9)	34(6.7)	41(8.1)	38(7.5)	0.882
	Female	56(11)	47(9.3)	36(7.1)	42(8.3)	35(6.9)	33(6.5)	
Handedness	Right	109(21.5)	89(17.6)	71(14)	70(13.8)	74(14.6)	68(13.4)	0.738
	Left	5(1)	5(1)	5(1)	6(1.2)	2(0.4)	3(0.6)	

**Table 5:** Demographic characteristics of SEN students.

		SEN students (n = 84) n(%)						p-value
Grade		1	2	3	4	5	6	
N		8	16	27	13	5	15	
Gender	Male	8(9.5)	12(14.3)	19(22.6)	12(14.3)	4(4.8)	9(10.7)	0.103
	Female	0(0)	4(4.8)	8(9.5)	1(1.2)	1(1.2)	6(7.1)	
Handedness	Right	0(0)	1(1.2)	1(1.2)	1(1.2)	1(1.2)	0(0)	0.539
	Left	8(9.5)	15(17.9)	26(31)	12(14.3)	4(4.8)	15(17.9)	

**Table 6:** The age range of the students in different grades.

	Grade						
	1	2	3	4	5	6
N	122	110	103	89	81	86
Average of age	6.98	8.07	9.09	10.17	11.15	12.12
SD of age	0.43	0.53	0.53	0.67	0.68	0.57
Minimum of age	6.17	7.13	8.22	9.15	10.13	11.30
Maximum of age	9.32	10.35	10.69	13.22	14.63	13.92

IBM SPSS Statistics 25 software (SPSS Inc. Chicago, IL, USA) was adopted in the statistical analyses and the statistical significance was set to p < 0.05 in all of the analyses. Two step-wise linear regressions were conducted to explore potential handwriting performance parameters contributing to the degrees of slant and tilt. The parameters to be entered into the linear regression model were shown in Table 2 as well as tilt for slant model and slant for tilt model. Before the linear regression, correlation analysis between the parameters was calculated in order to examine whether there were any covariates and potential confounds. All variables were calculated using the tablet’s recorded data.

## Results

The results of the stepwise linear regression between students’ Chinese handwriting slant/tilt degree and other features are shown in Table 7. The correlations between variables are shown in Table 8.

**Table 7:** Regression model of students’ Chinese handwriting slant/tilt degree and other features.

Dependent variable	Predictor	Standardized Beta	t	p-value
Slant	Tilt	-0.149	-3.671	0.000
	Gender	-0.088	-2.153	0.032
	SD_Pressure	0.082	2.010	0.045
Tilt	Ground_Time	0.250	6.366	0.000
	Slant	-0.147	-3.752	0.000
	SEN	0.089	2.267	0.024

**Table 8:** Pearson correlation between variables.

	Tilt	Slant	Age	Gender	Grade	Handedness	SEN	Ground_Time	Air_ Time	Air_ Ground_Ratio	Speed	SD_Time_Per_ Word	Pressure	SD_ Pressure
Slant	-0.157**													
Age	-0.172**	-0.012												
Gender	0.04	-0.103**	-0.006											
Grade	-0.17**	-0.005	0.953**	0.022										
Handedness	0.064	0.037	-0.016	-0.033	-0.008									
SEN	0.095*	0.028	0.047	-0.189**	0.039	-0.006								
Ground_Time	0.261**	-0.048	-0.599**	-0.045	-0.638**	-0.01	0.04							
Air_Time	0.207**	-0.067	-0.65**	-0.023	-0.678**	0.029	0.015	0.678**						
Air_Ground_Ratio	0.001	-0.035	-0.242**	0.019	-0.25**	0.044	-0.002	-0.117**	0.572**					
Speed	-0.214**	0.071*	0.744**	0.036	0.8**	-0.017	-0.063	-0.749**	-0.811**	-0.346**				
SD_Time_ Per_Word	0.208**	-0.022	-0.628**	-0.092*	-0.641**	0.049	0.084*	0.679**	0.812**	0.368**	-0.746**			
Pressure	0.091*	-0.088*	-0.057	0.206**	-0.065	0.036	-0.1**	0.066	-0.035	-0.127**	-0.043	-0.03		
SD_Pressure	-0.057	0.1**	-0.153**	-0.109**	-0.168**	0.018	0.083*	0.056	0.192**	0.151**	-0.131**	0.214**	-0.262**	
Out_Grid	0.023	0.008	-0.299**	-0.229**	-0.298**	-0.003	0.173**	0.25**	0.202**	-0.034	-0.224**	0.271**	-0.032	0.059

*: p<0.05

**: p<0.01.

Slant was found to be significantly correlated to Tilt, Gender and SD_Pressure. Tilt was found to be significantly correlated to Ground_Time, Slant and SEN.

To examine whether there was any moderating effect in the linear regression model, we also added the product of each pair of the predictors included the stepwise linear regression models for slant and tilt. The revised model is presented in Tables 9, 8 and 10. In the revised model, slant was found to be significantly correlated to Tilt×SD_Pressure and Gender. Tilt was found to be significantly correlated to Ground_Time, Slant and Slant×SEN.

**Table 9:** Revised regression model of students’ Chinese handwriting slant degree and other features.

Dependent variable	Predictor	Standardized Beta	t	p-value
Slant	Tilt×SD_Pressure	-0.163	-4.010	0.000
	Gender	-0.088	-2.322	0.000
Excluded variables	Tilt	0.031	0.158	0.874
	SD_Pressure	0.058	1.403	0.161
	Tilt×Gender	-0.049	-0.679	0.497
	Gender×SD_Pressure	0.260	1.107	0.269

**Table 10:** Revised regression model of students’ Chinese handwriting tilt degree and other features.

Dependent variable	Predictor	Standardized Beta	t	p-value
Tilt	Ground_Time	0.250	6.366	0.000
	Slant	-0.147	-3.752	0.000
	Slant×SEN	0.089	2.317	0.021
Excluded variables	Ground_Time×SEN	-0.061	-0.753	0.452
	Ground_Time×Slant	0.381	0.486	0.627
	SEN	-0.745	-0.944	0.346

Therefore, we rejected H_0_ and accepted H_1_ because there was sufficient evidence in the sample to show that there is a relationship between slant/tilt and other handwriting and demographic features of students.

## Discussion

We introduced a method of measuring and evaluating the slant and tilt of Chinese handwriting. We used this method to assess the manuscripts of primary school children. One advantage of our proposed slant and tilt measurement is that it was obtained by analyzing many different Chinese characters, giving us enough horizontal and vertical strokes to measure the slant and tilt reliably, even if the students wrote some characters incorrectly. As the computerized measurement was conducted by a handwriting tablet system, it was more objective and microcosmic than most slant/tilt evaluation methods based on hardcopy data.

This method enabled us to further analyse the students’ manuscripts by slant/tilt degree and to investigate the relationship between slant/tilt and other handwriting performance parameters. Our results in primary school students support the findings of Leung et al. [9] in adults. Their study showed that horizontal strokes in adults’ handwriting are commonly sloped upward and influenced by writing speed. This is consistent with our finding of a positive correlation between ground time and tilt value (negative tilt degree represents upward sloping and positive tilt degree represent downward sloping as shown in Fig 6). One explanation may be that writing horizontal strokes with a right up tilt decreases the friction between the paper and pen tip, thereby decreasing writing ground time. In particular, the findings from studies on this topic show that children tend to adopt several changes in their writing, presumably making it more efficient and consequently faster [28]. Moreover, when a writer writes a 0° horizontal line, a muscle in charge of deviation is employed but when a writer writes a slightly upward line, an extensor muscle is exerted. Since the extensor muscle is more powerful than the deviation muscle, the ground time will be reduced when a slightly upward stroke is written rather than an absolute horizontal stroke.

No significant correlation above 0.85 has been found between any variables is found. Therefore, it is rational to do stepwise linear regression without specifically removing any variables.

Slant and tilt are mutually important predictors to each other and they are negatively correlated. This means that when a student writes a character while disobeying the rule “horizontal strokes should be level and vertical strokes should be straight”, the resulting character shape would be sheared from a rectangle to a parallelogram, instead of simply rotated.

It is worthwhile to note that SEN is a significant parameter to influence tilt. Tilt is positively related to SEN, which implies SEN students tended to write less upward horizontal strokes than typically developed students. As mentioned in the previous paragraph, slant itself is an influential predictor to tilt. However, this effect is somehow offset by SEN, as the standardized beta for slant is negative and the standardized beta for slant×SEN is positive. This is due to the fact that typically developed students would adjust their horizontal strokes’ sloping to decrease ground time and to increase their writing speed. Habitual handwriting changes have also been found in studies of other cross-sectional and longitudinal designs [29][30]. However, SEN students tend to stay in the way they were taught to write the strokes for the first time. For instance, some SEN students may treat absolute horizontal strokes and upward strokes as different types of strokes. They may think that it is wrong and unacceptable to write horizontal strokes slightly upwards because they stick to inflexible routines and overlook the details. A lack of appropriate upward tilt can be an indicator for screening SEN students. This also indicates that SEN students may need a special reminder to change their handwriting more freestyled to increase their writing speed.

Regarding slant, gender and SD_pressure are significant predictors. The finding that SD_pressure is positively related to slant degree means that students whose pen pressure fluctuates tend to slant to the right as shown in Fig 6 during the writing process. It is due to the fact that when slanting to the left or vertically in their writing, the writers need to shift their wrists while when slanting to the right they just need to shift their fingers. As the wrist can provide more steady support to the pen than fingers, the variation of pen pressure during writing is smaller. It can be inferred that students who tend to slant to the right may have difficulty to control their wrists, as slanting to the right is not a good practice in Chinese handwriting. Therefore, slanting to the left can be an indicator of the potential neuromuscular disorder as it reflects less use of wrist [31]. This is also evidenced that tilt×SD_Pressure a stronger predictor than tilt and SD_Pressure individually, as lack of wrist agility influence slant, tilt and SD_Pressure at the same time. In respect of gender, some researches indicated that more boys are affected by neuromuscular disorders than girls [32], which could also suggest that gender could be a significant predictor for slant values.

Grade or age is not a significant predictor for slant and tilt level. As Simner [33][34] indicated that the established patterns learned at home, in kindergarten, or in preschool may be difficult to change through formal handwriting instruction in schools. Therefore, whether an instruction for children in higher grades can help them keep their horizontal strokes from tilting requires further investigation.

Whether the slant/tilt level of one’s handwriting should be treated as a type of error influencing legibility or merely a personalized handwriting style must be considered. Several studies have confirmed that children’s scripts become more personalized and varied during the upper-elementary grades. Specifically, more students combined cursive writing into the manuscripts they were taught in Tarnopol and Feldman [35]. The spatial variability of stokes within letters increased considerably between grades 4 and 5 in Meulenbroek and van Galen [36]. Furthermore, older children used less fixed anchoring when joining two strokes than younger children in Nihei [37]. These findings reveal that students’ handwriting becomes more freestyled and departs from typical handwriting instruction with age. However, it does not necessarily mean that their handwriting ability deteriorates, as their writing speed increases steadily. The handwriting product also turns into more regular and smoother shapes in higher-grade students [38]. We should also consider a proper slant and tilt range for students’ handwriting to match with the general guideline that “horizontal strokes should be level and vertical strokes should be straight.” This does not necessarily mean 0° for horizontal strokes and 90° for vertical strokes. Specifically, some of the students were observed to demonstrate smooth handwriting techniques with a certain slant/tilt degree.

This study had several limitations. First, approximately 33% of the parents refused to participate and some parents refused to reveal the specific SEN the students had. Therefore, attrition bias exists in this study. Due to such recruitment difficulties, the students who did participate may not fully represent the handwriting performance of Hong Kong primary school students. Since this recruitment is only conducted in public primary schools, the socio-economic factors between public and private school students cannot be reflected in this study. Although there may be differences in the learning curricula and handwriting practices of parents, sampling in different districts across Hong Kong should have minimized these differences. Second, a few limitations were related to the Digital Note tablet. Although its reliability has not been investigated, other similar handwriting tablets have been validated to support its reliability. It is suggested that the reliability of the Digital Note tablet can be established by examining the correlation of its results with other handwriting tablets. As this study was only validated in primary school students, the current findings must be generalized to children in kindergarten and higher grades with caution. In our future work, analyses between slant/tilt level and other handwriting product parameters will be performed to investigate the potential relationships between and reasons for students’ handwriting results.

## Conclusion

Our results demonstrate the relationship between tilt level and Chinese handwriting performance in primary school children. Tilt and slant were negatively correlated with each other, showing a shearing reshape in students’ handwriting. Apart from this, the tilt level in the students’ Chinese handwriting was positively related to ground time and slant×SEN, while slant is negatively related to the tilt×standard deviation of pen pressure and female gender. These findings suggest slant and tilt can be adopted as an indicator in students’ SEN diagnosis. If a proper pedagogy can be developed based on these findings, it can facilitate SEN students to increase their writing speed.
